# Electrode impedance analysis of chronic tungsten microwire neural implants: understanding abiotic vs. biotic contributions

**DOI:** 10.3389/fneng.2014.00013

**Published:** 2014-05-08

**Authors:** Viswanath Sankar, Erin Patrick, Robert Dieme, Justin C. Sanchez, Abhishek Prasad, Toshikazu Nishida

**Affiliations:** ^1^Electrical and Computer Engineering Department, University of FloridaGainesville, FL, USA; ^2^Biomedical Engineering Department, University of MiamiCoral Gables, FL, USA

**Keywords:** chronic neural implants, electrode impedance, corrosion, insulation delamination, finite element modeling

## Abstract

Changes in biotic and abiotic factors can be reflected in the complex impedance spectrum of the microelectrodes chronically implanted into the neural tissue. The recording surface of the tungsten electrode *in vivo* undergoes abiotic changes due to recording site corrosion and insulation delamination as well as biotic changes due to tissue encapsulation as a result of the foreign body immune response. We reported earlier that large changes in electrode impedance measured at 1 kHz were correlated with poor electrode functional performance, quantified through electrophysiological recordings during the chronic lifetime of the electrode. There is a need to identity the factors that contribute to the chronic impedance variation. In this work, we use numerical simulation and regression to equivalent circuit models to evaluate both the abiotic and biotic contributions to the impedance response over chronic implant duration. COMSOL® simulation of abiotic electrode morphology changes provide a possible explanation for the decrease in the electrode impedance at long implant duration while biotic changes play an important role in the large increase in impedance observed initially.

## Introduction

Tungsten micro-wire electrode arrays continue to be used as chronic implants for single-unit neuronal recording (Williams et al., 1999a,b; Rennaker et al., 2005; Rizk et al., 2009; Freire et al., 2011). Implanted electrodes in general suffer recording performance degradation over time (Williams et al., 1999a; Ward et al., 2009). Understanding the reasons for poor recording performance over time is integral for the adaptive design of future electrode arrays. For tungsten micro-wire electrodes, structural modification of the recording site by corrosion and insulation delamination (Sanchez et al., 2006; Patrick et al., 2011; Prasad et al., 2012; Streit et al., 2012) as well as tissue encapsulation due to the foreign-body immune response (Szarowski et al., 2003; Biran et al., 2005, 2007; McConnell et al., 2009) are known to occur. Although it is known that both abiotic and biotic effects contribute to changes at the electrode recording site, it is still unclear to what extent each effect has on the chronic recording performance of the electrode.

Impedance is a metric commonly used to assess electrode performance. Chronic *in-vivo* studies (Prasad and Sanchez, 2012) have confirmed a functional correlation between the electrode impedance value at 1 kHz and the overall neuronal yield during the implanted duration. It was observed in that study that low array yields were associated with very low impedance values or very high impedance values, and the best array yield was observed for an impedance range of 40–150 kΩ at 1 kHz for the implanted 50 μm diameter blunt-cut tungsten micro-wires. The electrode impedance also varied over time increasing during the first few weeks of implantation followed by a drop in the impedance value in the latter phase of the implant duration. Though these observations suggest that the electrode impedance is affected by some short-term and long-term factors, the underlying driving mechanisms are not fully understood. Furthermore, the impedance variation for tungsten microwires itself varied across different implanted animals.

The working model is that network analysis of the complex impedance spectra of implanted electrode arrays may yield useful information about the changes occurring in the vicinity of the electrode recording sites in the neural tissue. This is supported by the results of Williams et al. who showed a distinction between severe and nominal inflammatory response to tungsten electrodes implanted in an animal model for 19 days by comparing the complex impedance spectra (Williams et al., 2007). Nyquist plots were used to show the progression of the inflammatory response over time. However, the effect of corrosion or insulation delamination was not considered in their paper.

In this paper, we analyze the complex impedance spectra of tungsten micro-wire electrodes that were previously implanted for 9-months into the rat somatosensory cortex. We show progress toward decoupling the abiotic (e.g., recording-surface structural modification) effects from the biotic (e.g., tissue encapsulation) effects with the analysis of the complex impedance spectra of the implanted electrodes. Graphical comparisons as well as regression to an equivalent circuit model provide qualitative and quantitative results. Finite element analysis package COMSOL® Multiphysics® (Burlington, MA) is used to simulate abiotic effects of different electrode surface variations on the impedance. We used pre-implant and post-explant SEM imaging and surface roughness analysis to provide evidence of abiotic structural changes.

## Materials and methods

### Analysis of *in-vivo* electrode array data

#### Electrode array

Sixteen-channel micro-wire arrays [Tucker-Davis Technologies (TDT), Alachua FL] were used for this study. The microwires were 50 μm in diameter, 5 mm long, and blunt cut using a laser beam. The tungsten wires were plated with a thin film of gold of thickness ~2–5 μm and insulated with a layer of polyimide of approximate thickness 10 μm. The wires were positioned in a 2 × 8 configuration with spacing of 250 μm between two adjacent microwires.

#### Implantation and recording

The electrode array was implanted in the somatosensory cortex of an adult male Sprague-Dawley rat. Aseptic surgical techniques were used for the implantation procedure. All procedures were approved by the Institutional Animal Care and Use Committee, University of Miami, FL. Electrophysiological recordings were made on the animal three to four times a week and each recording session lasted approximately 20 min. A custom testbed was developed that allowed the animal to move freely during the recording session. Impedance was measured before every recording session on all 16 microwires using a small current (maximum 1.4 nA) that did not affect the electrode properties. The surgical and recording procedures are described in detail in Prasad and Sanchez (2012). The structural changes were investigated by imaging the electrodes surfaces before and after the implant.

#### Characterization of structural changes

The structural changes in the implanted electrodes were studied by characterizing the microwire arrays through qualitative methods using scanning electron microscope (SEM) imaging and quantitative methods via laser scanning microscope surface roughness measurements. Tungsten microwires were imaged before and after explantation using SEMs (CarryScope SEM, JEOL, Inc., and FEI XL-40 field emission gun SEM). The imaging procedure is described in detail in Prasad et al. (2012).

The pre- and post-implant SEM images of the electrode provided only a qualitative idea of the variation in the surface morphology. In order to quantitatively assess the surface roughness and corrosion depth of the implanted electrodes, some of the arrays were analyzed for surface roughness using a laser scanning microscope (Keyence VK-9700 Color 3D Laser Scanning Microscope) at the Materials Evaluation and Testing Laboratory, South Dakota State University. The depth profile of the wires in the array was measured and the average height with respect to the reference line was calculated.

#### Impedance measurement and analysis of magnitude at 1 kHz

The NanoZ® impedance tester (TDT, FL) was used to measure the daily *in-vivo* impedance values. The NanoZ applies a constant voltage of 4 mVpp sinusoidal waveform which results in a maximum test current of 1.4 nA. Compared to an estimated corrosion current on the order of 100 nA at equilibrium from results in Patrick et al. (2011), the 1.4 nA current used for the impedance measurements will negligibly affect the normal corrosion processes. The impedance measurement process involved 40 cycles of measurement on each microwire and an average value was calculated to minimize measurement errors. The impedances were recorded at 1, 2, 5, 10, 20, 50, 100, 200, 500 Hz, 1 and 2 kHz. Prior to implantation, the impedance of each microelectrode was characterized in 0.9% phosphate buffered saline (PBS) that served as a baseline for the *in vivo* measurements. In the case of *in vitro* pre-implant testing, the reference was made to a stainless steel ground wire connected to the MEA assembly and dipped in the same saline solution, whereas for *in vivo* chronic testing, the reference was made to the same stainless steel ground wire connected to a stainless steel bone screw drilled into the skull.

From the measured *in vivo* impedance data, the percentage change in impedance was calculated for each individual wire and averaged for all wires for the implanted duration. The impedance value measured on the surgery day was considered as the reference value and the percentage change was calculated for this reference. Since the temporal variation of the *in vivo* impedance approximately follows a Gaussian profile, the calculated percentage change in impedance values were fitted with a Gaussian curve using the built-in curve fitting tool in OriginPro® (OriginLab Corporation, Northampton, MA) given by

(1) $$y=y_{0}+\frac{A}{w\sqrt{\pi /2}}e^{-2\frac{{\left(x-x_{c}\right)}^{2}}{w^{2}}}$$

where, *w* is the standard deviation, *x* is the individual calculated value, *x*_*c*_ is the average, and *A* and *y*_0_ are constants. Percentage change higher than 600% were treated as outliers and were not included in the calculations.

### Graphical analysis and regression of complex impedance data

The real and imaginary parts of the complex impedance data as a function of frequency were plotted with logarithmic scales on both axes to capture both low as well as high frequency data. These complex-plane impedance plots, compared to Nyquist or Bode plots, can better identify physical processes (Orazem et al., 2006) and make trends in the temporal changes more apparent. Eight timestamps were chosen to adequately capture the temporal impedance trend, day 1 of implant and days 7, 15, 31, 60, 106, 149, and 205 post implant.

**Figure 1:**
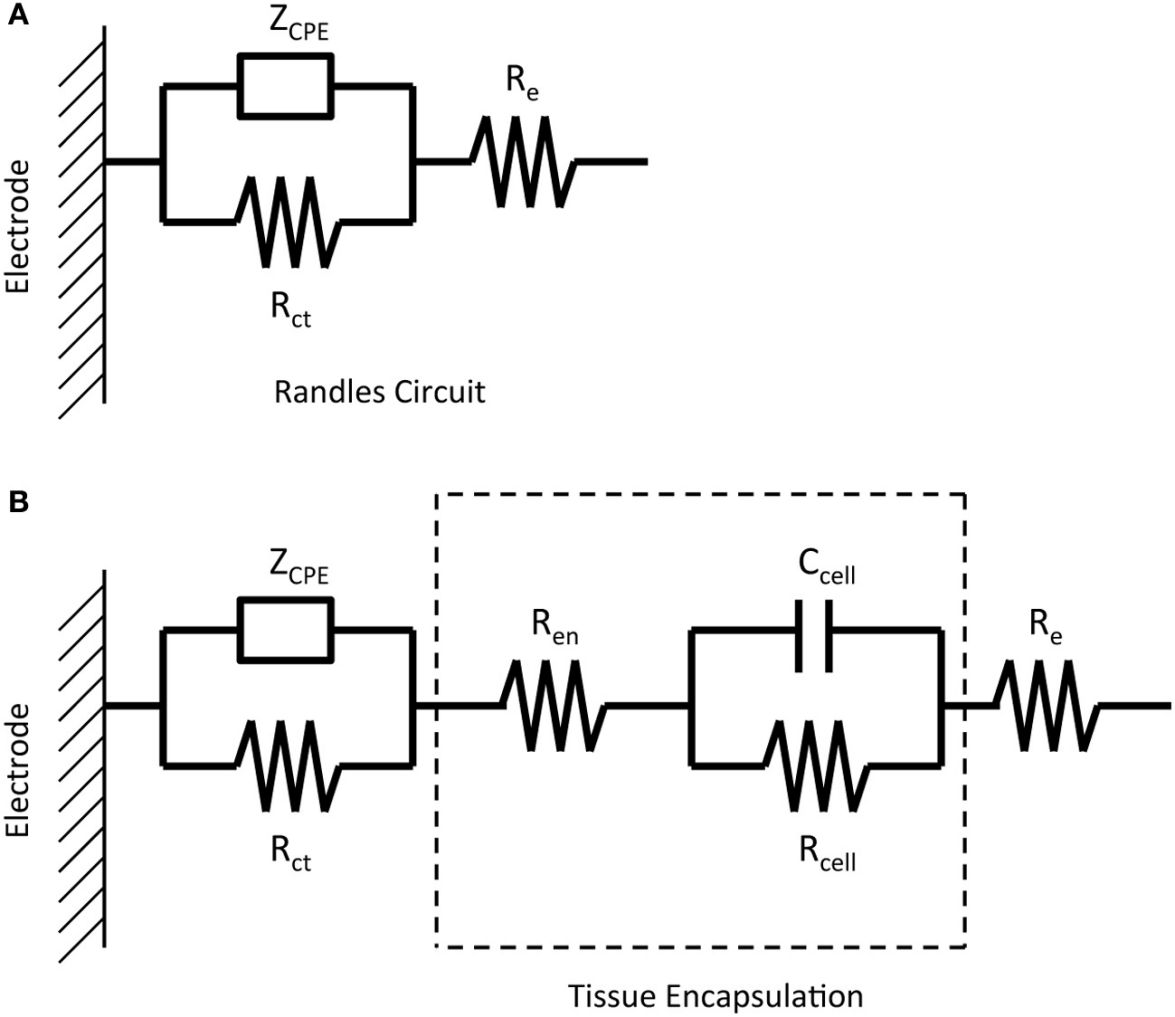
**Equivalent circuits representing the impedance of a micro-wire without the presence of tissue encapsulation due to the inflammatory response (A) and with the presence of tissue encapsulation (B)**.

Regression of the complex impedance data was performed using the non-linear least squares regression function in Matlab. The real and imaginary parts of the impedance spectrum were fit to one of two equivalent circuits shown in Figure 1. The circuit in Figure 1A, commonly known as the “Randles circuit,” models the electrochemical interface of a bare metal electrode in an electrolyte neglecting the diffusion related Warburg impedance (Brug et al., 1984; McAdams et al., 1995). *R*_*ct*_ is the charge transfer resistance, which models the resistive pathway due to electrochemical reactions (i.e., tungsten corrosion), and *Z*_*CPE*_ is the constant phase element impedance given by

(2) $$Z_{CPE}=\frac{1}{{\left(j\omega \right)}^{\alpha }Q}$$

where ω is the frequency, α is a number from 0 to 1, and *Q* is the constant phase element (CPE) coefficient with units s^α^/Ω cm^2^. The CPE element is used to model the capacitive double layer at the electrode interface. The CPE, rather than an ideal capacitor, better accounts for non-idealities due to surface inhomogeneity (Brug et al., 1984) and is commonly used in electrode/electrolyte equivalent circuit models (McAdams et al., 1995; Johnson et al., 2005; Otto et al., 2006; Williams et al., 2007; Lempka et al., 2011). *R*_*e*_ models the resistance of the electrolyte seen by the electrode and is also referred to as the spreading resistance to a distant ground. Figure 1B adds a simplified circuit that models the tissue encapsulation layers through a series resistor *R*_*en*_ and parallel combination of a resistor *R*_cell_ and capacitor *C*_cell_. (Johnson et al., 2005; Otto et al., 2006; Williams et al., 2007; Lempka et al., 2011). The tissue encapsulating the electrode due to the immune response consists of layers of extracellular matrix proteins (e.g., collagen, fibronectin, and laminin) and reactive glial cells and macrophages (Grill and Mortimer, 1994; Kim et al., 2004). *R*_*en*_ models encapsulation by extracellular matrix proteins, and *C*_cell_ and *R*_cell_ model the resistive and capacitive pathways through the cellular layer, respectively.

**Figure 2:**
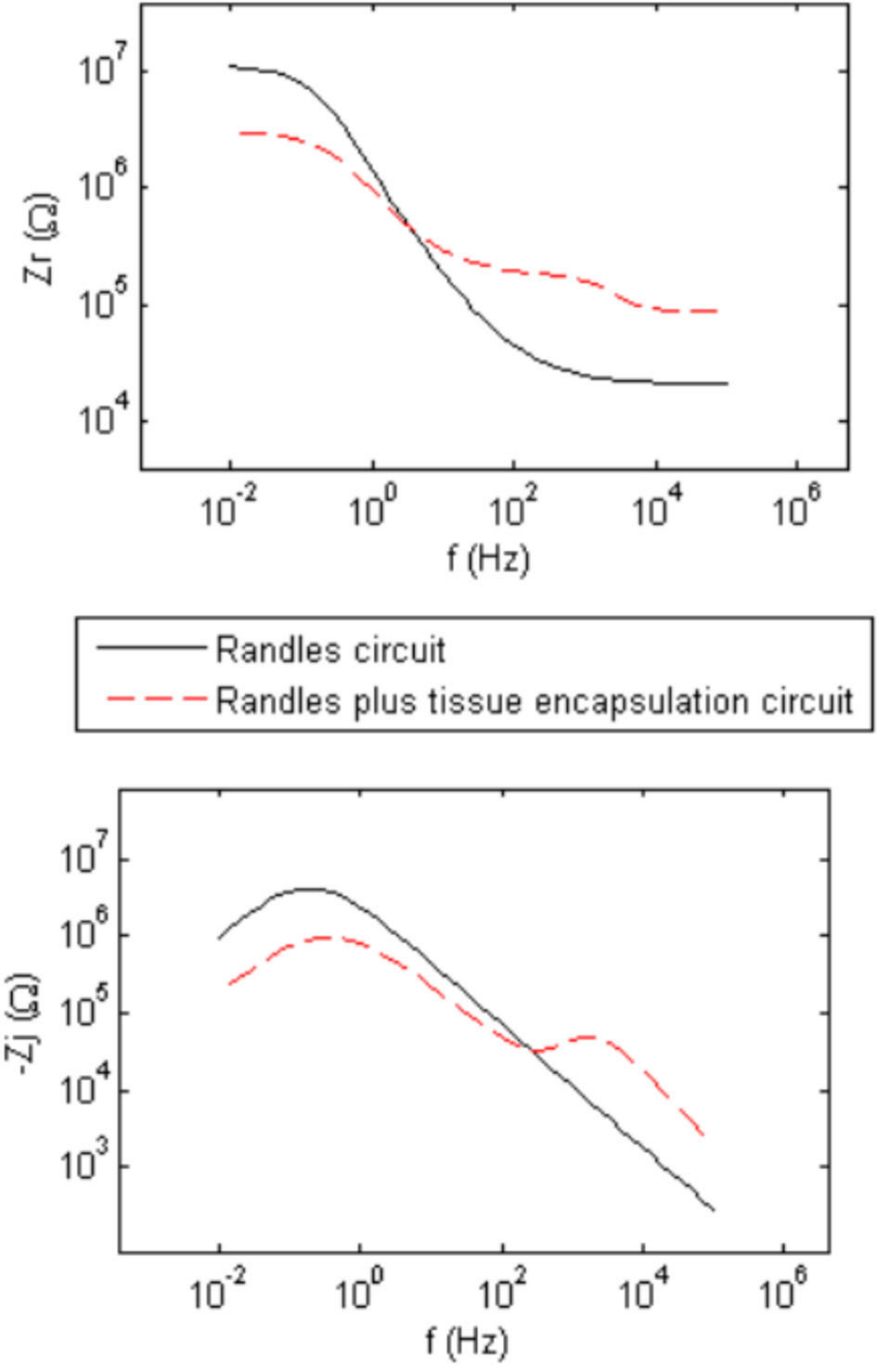
**Generic calculated real (*Z*_*r*_) and imaginary (*Z*_*j*_) parts of the complex impedance as a function of frequency representing the two equivalent circuits in Figure 1 having abiotic only and abiotic and biotic components which illustrate the presence of one peak for the Randles circuit and two peaks for the Randles combined with Tissue Encapsulation circuits for the imaginary impedance**.

Figure 2 shows generic complex-plane impedance plots calculated using the two equivalent circuits in Figure 1. One distinguishing feature of the imaginary impedance as a function of frequency is the presence of only one peak for the Randles circuit and the presence of two peaks in the circuit that adds tissue encapsulation components to the Randles circuit. These peaks occur at the frequencies given by the reciprocal of the RC time-constants in the circuit. *R*_*ct*_ and *Z*_CPE_ determine the low-frequency peak and the high-frequency peak is determined by the adherent cellular components, *C*_cell_ and *R*_cell_. Thus, the presence of a cellular biotic layer at the electrode surface may be inferred by examining the imaginary impedance data. The real impedance as a function of frequency provides information on the electrolyte and encapsulation resistance, *R*_*e*_ and *R*_*en*_, at high frequencies where the impedance of CPE and *C*_cell_ are small compared to the parallel resistive components. The high-frequency asymptotic value converges to the electrolyte resistance *R*_*e*_ in the Randles circuit and the addition of *R*_*e*_ and *R*_*en*_, for the circuit that includes encapsulation components. We use these graphical impedance analysis techniques to assess the factors causing temporal variation of impedance for our chronically implanted electrode array.

### COMSOL® finite element analysis (FEA)

**Figure 3:**
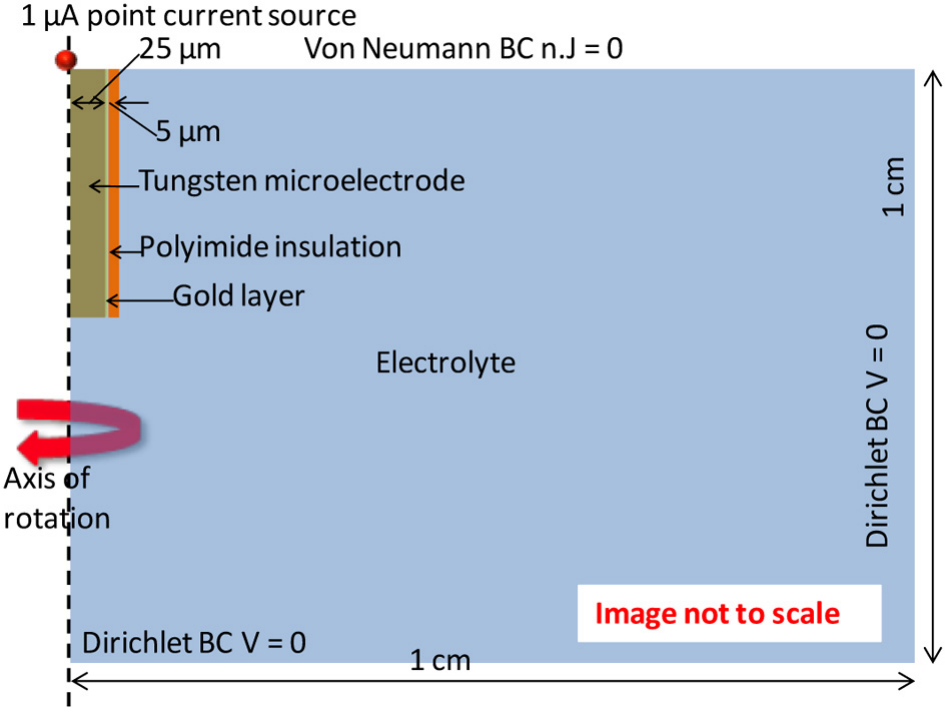
**Illustration of the electrode-electrolyte interface and the boundary conditions used in the COMSOL® finite element model**.

Finite element analysis of the electrode-electrolyte interface was made in COMSOL® Multiphysics software package to investigate how the electrolyte resistance, *R*_*e*_, changes over time due to simulated structural modifications. The tungsten electrode was modeled using a three-dimensional (3-D) geometry consisting of a 50 μm thick tungsten microwire surrounded by 1 μm thick gold layer and insulated with 5 μm thick polyimide insulation surrounded by a region of cerebrospinal fluid (CSF) electrolyte. The COMSOL® AC/DC module (or electric current physics) was selected to numerically solve the governing physics. The electrolyte boundaries were extended up to 1 × 1 cm to satisfy the semi-infinite boundary condition. A point current source of 1 μA was placed at the center of the top (cortex boundary) surface of the electrode. Figure 3 shows an illustration of the COMSOL® finite element 3-D model of the electrode-electrolyte interface. The electrode was assumed to be a uniform conductor; hence the potential distribution across the electrode is constant. A Dirichlet boundary condition of *V* = 0 was assumed at the electrolyte boundaries since the boundaries are supposed to be semi-infinite, while a von Neumann boundary condition of $\vec{n}\;\cdot \;\vec{j}=0$ was assumed at the cortex boundary, which ensured electrical insulation at the boundaries. The conductivities of the different materials used for the model are given in Table 1. Tetrahedral elements with size ranging from 1 to 400 μm were used for meshing the domains.

**Table 1:** **Conductivity values used for the COMSOL® finite element model**.

**Material/layer**	**Conductivity (S/m)**	**References**
Tungsten	18.94 × 10^6^	Serway, 1998
Gold	40.98 × 10^6^	Serway, 1998
Polyimide	1 × 10^-16^	HD Microsystems, 2001
Cerebrospinal fluid	1.8	Baumann et al., 1997

## Results

### *In vivo* experimental results

**Figure 4:**
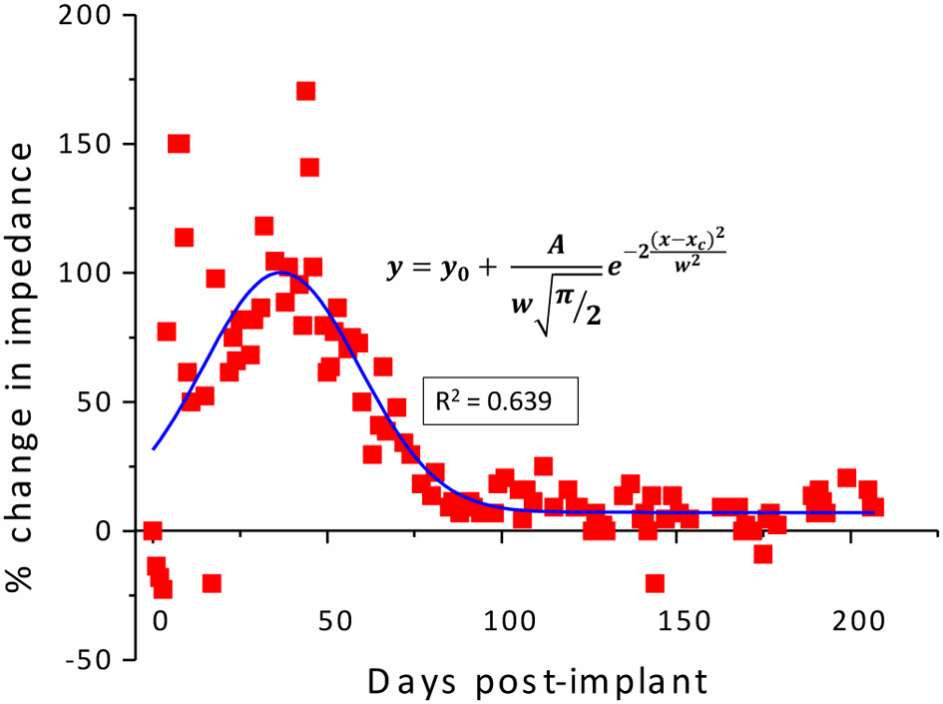
**Average percentage change in the *in-vivo* impedance averaged for all wires of electrode array R9 plotted against the implanted duration and fitted with a Gaussian curve**.

**Figure 5:**
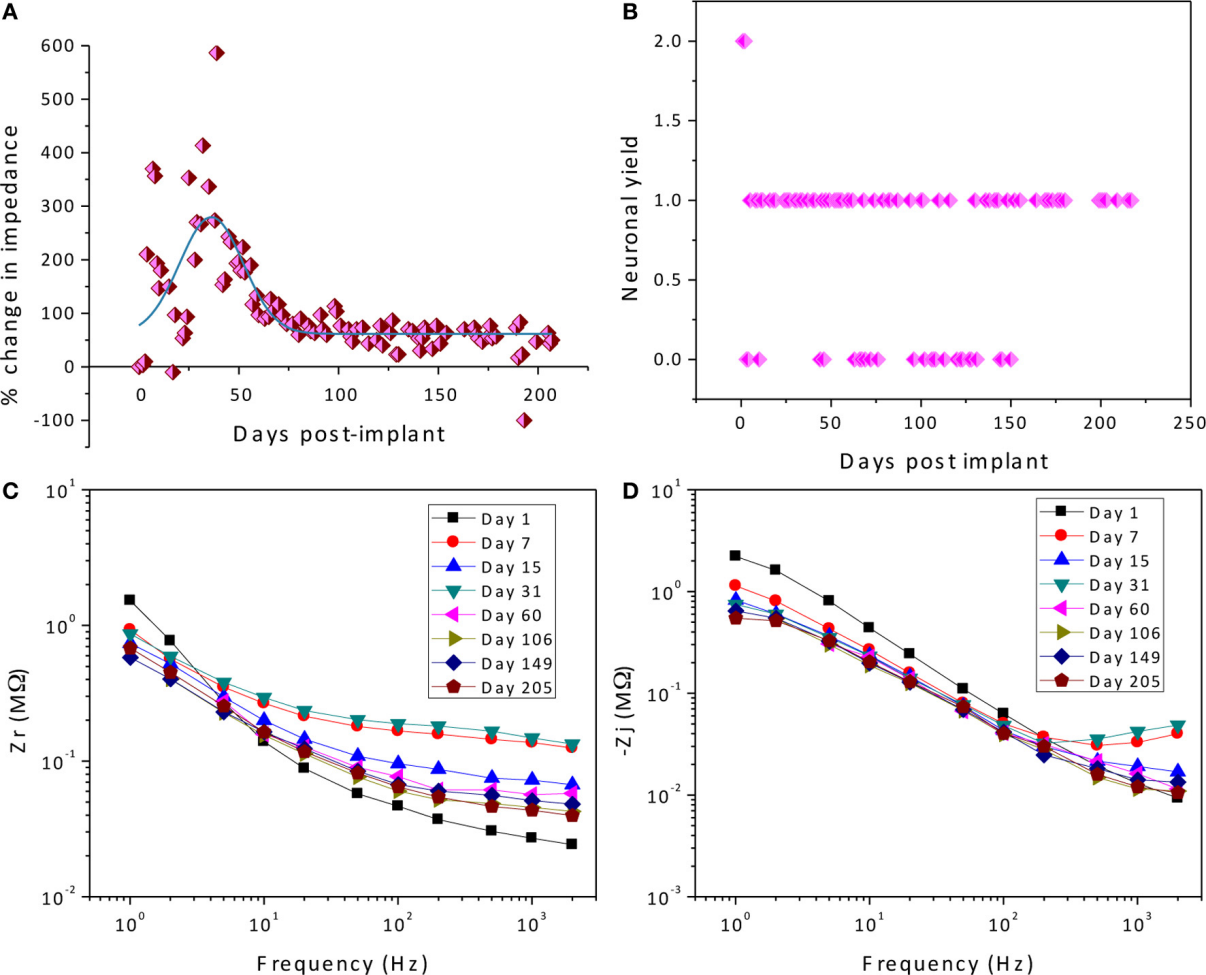
**Detailed data of the percent change in impedance magnitude at 1 kHz (A), neuronal yield data over time (B), complex-plane impedance plots over time (C,D) for wire 12**.

As described previously (Prasad et al., 2012), the electrode array under study was deemed a functionally optimal performing array having 50–70% of the wires functional during the extent of the implant (216 days). Also, the *in vivo* impedance magnitude measured at 1 kHz stayed within the 40–150 kΩ range determined optimal for functional electrophysiological recordings for this array type. The average temporal variation, averaged over 16 electrodes, is shown in Figure 4. The average change in impedance at 1 kHz follows a Gaussian profile. However since averaging can mask individual variation, it is also instructive to look at specific electrodes that highlight the variation between electrodes implanted at the same time in the same animal. Detailed data of the percent change in impedance magnitude at 1 kHz, complex-plane impedance plots over time, and neuronal yield data over time for one representative wire out of the array (wire 12) are shown in Figure 5. Data for all wires in the array are given in the supplemental materials.

**Figure 6:**
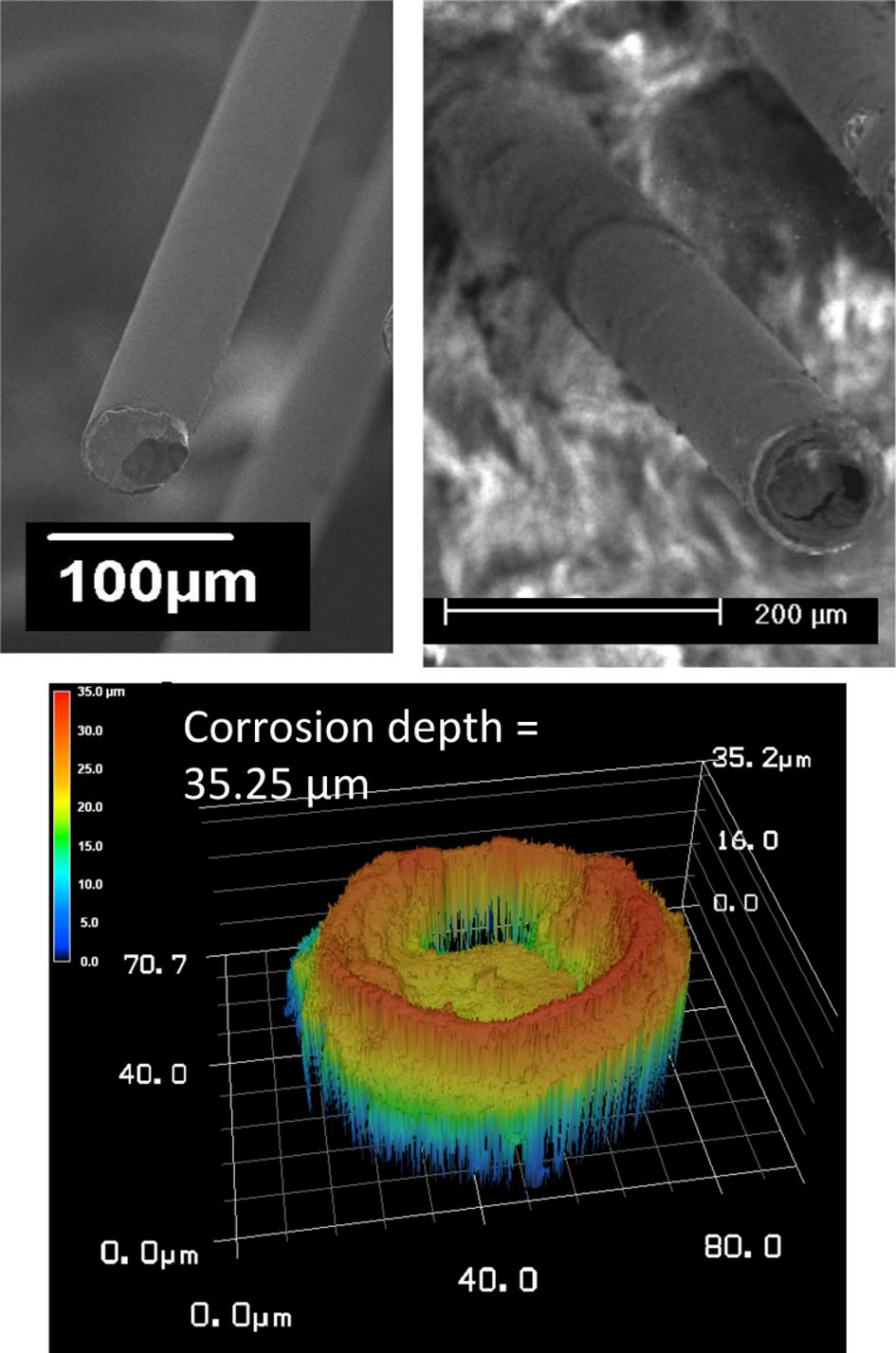
**SEM images of the recording site pre- and post-implant (top), and relief images of the surface showing a calculated corrosion depth (bottom) for wire 12**.

Corrosion of the tungsten and delamination and cracking of the polyimide insulation are abiotic factors that contribute to morphological changes at the electrode/tissue interface. SEM images of the recording site pre- and post-implant, and relief images of the surface showing a calculated corrosion depth for one wire out of the array (wire 12) are shown in Figure 6. Comparison between the pre-implant and the post-implant SEM images of the electrodes revealed similar amounts of tungsten corrosion in all the 16 wires. The tungsten core was recessed within the outer layers of gold and polymer insulation. The measured average corrosion depth for all of the wires was 38.8 μm with a standard deviation of 9.4 μm. Moreover, all wires had some variation of visible insulation damage including delamination and cracks. The majority of the damage was delamination at the electrode surface. In two electrodes out of the array, there were noticeable cracks in the insulation that reached, at maximum, 30 μm below the recording surface.

In all wires in the array, there were large variations in the impedance magnitude over time, with the largest variability occurring within the first few weeks. This large variability happens to occur when the abiotic and biotic factors are most in flux. For example, the rate of tungsten corrosion is larger within the first few weeks of implantation (Prasad et al., 2012), and the inflammatory response does not achieve its chronic stage until after 4–6 weeks (Turner et al., 1999). Thus it is unclear which factor, abiotic or biotic, contributes most to this trend.

We attempt to uncover the mechanisms for the temporal variations by examining the complex-plane impedance data for select days using the graphical analysis described in Graphical Analysis and Regression of Complex Impedance Data. Figure 5 shows a trend in the complex impedance seen in all wires of the array. The imaginary impedance as a function of frequency (subfigure D) shows two peaks instead of one on every day except for the first day suggesting the presence of a second RC time-constant after day one. These observations suggest a definite biotic component to the impedance magnitude at 1 kHz after day one, since the peak due to a cellular component in the complex impedance vs. frequency is apparent. Our hypothesis that these temporal variations in the electrode impedance have a stronger biotic contribution in the days following implant surgery is supported by the large cellular changes that occur around the microelectrodes due to initial foreign body response. Also the peak occurring at higher frequencies (>400 Hz) is more pronounced primarily on days 7, 15, and 31 after implantation, which is usually the period during which the tissue encapsulation layer due to the inflammatory response is forming (Turner et al., 1999). The second peak is less pronounced in the later days and the impedance values at the higher frequencies decrease. The real part of the impedance as a function of frequency (subfigure C) shows a similar trend in all of the wires of the array in which the impedance value at higher frequencies (>50 Hz) increases to a maximum in the beginning (days 7–31) and then decreases and remains more constant over time. These results are also consistent with our previously reported results where we observed the tissue inflammatory response to be reducing for long-term animals such as the one examined here (Prasad et al., 2012). Further, an abiotic factor such as insulation deterioration is also one of the likely causes of the reduction of impedance in the chronic phase. Circuit elements *R*_*e*_, and *R*_*en*_ are important factors in this trend and will shed more light on the influence of biotic and abiotic effects.

### Regression of complex impedance data

**Figure 7:**
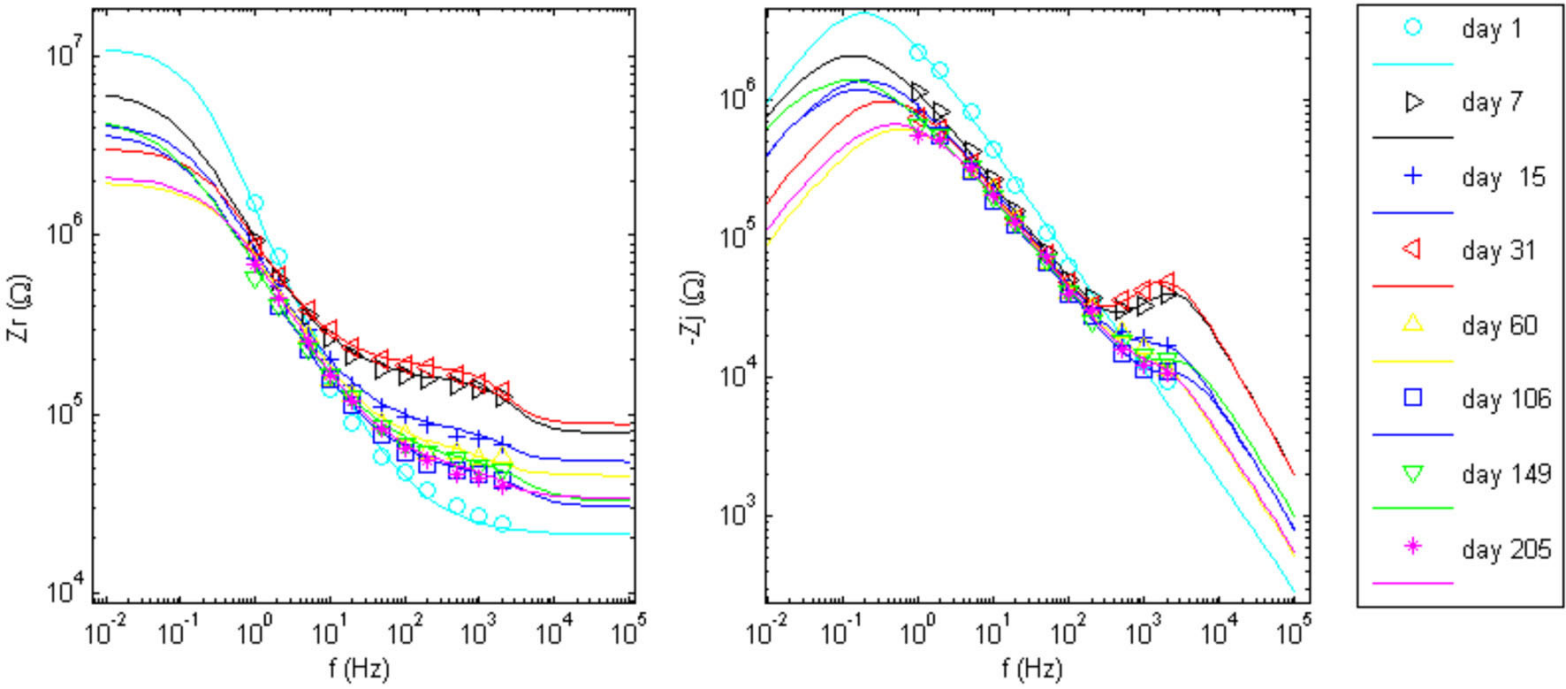
**Impedance data points and the regressed fit lines of complex impedance for wire 12 to the circuits shown in Figure 1**. Day 1 was fit to the Randles circuit, only. The equivalent circuit with added tissue encapsulation components are used to fit the data for all other days post implant.

The goal of the regression is to quantify each circuit parameter such that the abiotic and biotic contributions to the impedance magnitude at 1 kHz may be identified. One electrode in the array, wire 12 (complex impedance shown in Figures 5C,D), was chosen as a representative sample for regression analysis. The impedance for day 1, since it only has one time constant, was fit to the Randles circuit and the remaining data sets were fit to the circuit that includes the encapsulation components. In the regression, one resistor encompassing *R*_*e*_ and *R*_*en*_ was used in the equivalent circuit that incorporated encapsulation components. Figure 7 shows the regression results with fits to the limited data points.

Further analysis is needed to decouple the electrolyte resistance and the encapsulation resistance for all days after the first. The electrolyte impedance *R*_*e*_ is dependent on the surface morphology of the electrode. With corrosion and insulation deterioration occurring at the electrode surface, *R*_*e*_ will likely change over the implant time; however, the extent is not well understood. For example, what effect does a recessing electrode surface have on the electrolyte resistance? This question has not been addressed in the literature to the best of our knowledge. We rely on COMSOL numerical simulation to estimate the changes to *R*_*e*_ over time which is used to estimate a value for *R*_*en*_ (Discussion section).

### COMSOL® FEA results

The response of structural changes to the electrolyte resistance is analyzed in COMSOL®. Our model for electrode surface morphology was developed based on our knowledge obtained from prior *in vitro* (Patrick et al., 2011) and *in vivo* (Prasad and Sanchez, 2012) impedance studies. It comprises morphological changes to the metal, tungsten, via corrosion and to the polymer insulation via crack formation and delamination.

The results from the *in vitro* and *in vivo* studies suggest that the electrode will undergo continuous corrosion during the implantation period. However, the corrosion rate may be highly dynamic. The rate of corrosion is influenced by the presence of chemical species such as hydrogen peroxide (H_2_O_2_) (Patrick et al., 2011), which is produced by reactive microglia that surround the implanted microwire. The *in-vitro* corrosion study showed that gold plated tungsten wires corrode at the rate of 10,000–20,000 μm/year in PBS in the presence of millimolar concentrations of H_2_O_2_, while the rate drops to 300–700 μm/year in the absence of H_2_O_2_ (Patrick et al., 2011).

In the *in vivo* setting, the immune response gives an indication for how corrosion may evolve. We have shown the corrosion to be present in all microwires but the rate of corrosion was found to be accelerated in the first few weeks following electrode implant compared to chronic periods (Prasad et al., 2012). Coincidentally, there are larger changes that occur in the vicinity of the electrode recording tips due to the inflammatory response in the early hours to weeks following implant. The inflammatory response activates the microglial cells, which produce superoxides and add unknown concentrations of H_2_O_2_ to the CSF (Burke and Lewis, 2000). During the first few weeks of implantation, we assume there is a higher concentration of H_2_O_2_, which leads to a higher rate of tungsten corrosion. When the inflammation response progresses to the gliosis phase, the number of surrounding glial cells decreases, and the glial sheath becomes more compact (Polikov et al., 2005). Hence, we assume the rate of corrosion decreases because there is a smaller amount of H_2_O_2_ being produced by the remaining glial cells, and the dense glial sheath may impede diffusion of reacting species, thereby making the corrosion rate mass-transfer limited. Thus far, our model for temporal tungsten morphology is that the exposed tungsten will uniformly corrode (as compared to pitting corrosion) as soon at it is implanted with a rate that will increase with time up to formation of the compact glial sheath and then decrease to a constant value. These assumptions were made after close observation of electrode recording sites for these arrays for varying implant durations (acute to up to 9 months).

**Figure 8:**
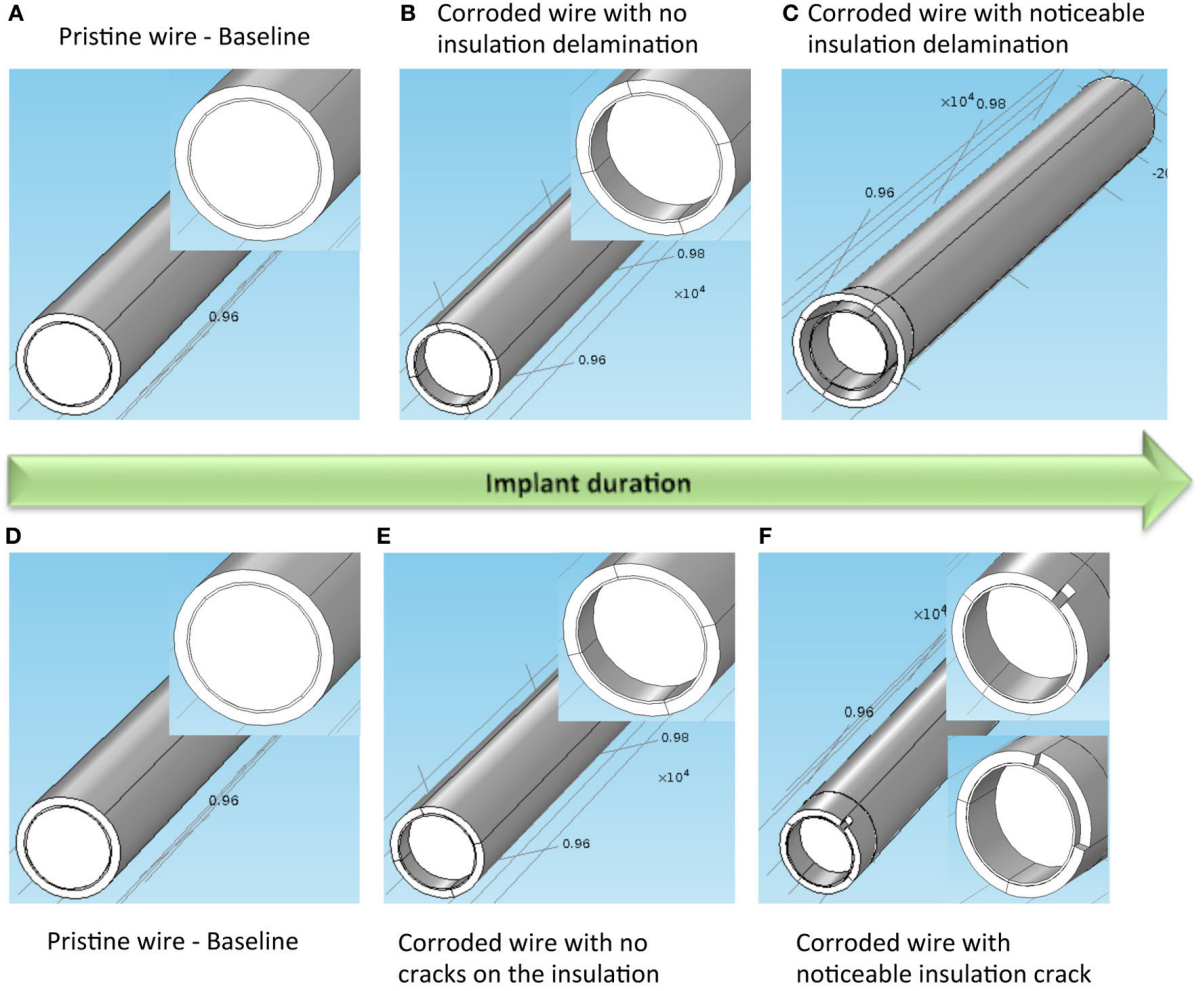
**Case 1 for modeled electrode surface modification over implant duration involving both corrosion and insulation delamination. (A)** Pristine electrode with intact metal and insulation, **(B)** electrode with corroded metal and no insulation delamination, and **(C)** electrode with corroded metal and noticeable insulation delamination. Inset on the left and middle images shows a closer view of the gold layer around the tungsten. Case 2 for modeled electrode surface modification over implant duration involving both corrosion and insulation cracking. **(D)** Pristine electrode with intact metal and insulation, **(E)** electrode with corroded metal and no insulation crack, and **(F)** electrode with corroded metal and noticeable insulation crack. Inset on the left, middle and right images shows a closer view of the gold layer around the tungsten.

The effect of two cases of electrode surface variations on the electrolyte impedance *R*_*e*_ were studied in detail. In both cases, corrosion is assumed to occur while in Case 1, the added variation due to insulation delamination/peeling is modeled and in Case 2, the added variation of insulation cracking is considered. Figure 8 illustrates the model geometry for electrode surface modification over implant duration involving both corrosion and insulation delamination (Case 1) and both corrosion and insulation cracking (Case 2), respectively.

In Case 1, the insulation delamination was modeled as a gap between the electrode and the insulation (Figure 8A). Impedance was calculated at seven different instances with each instance representing a different surface. The first instance was a pristine wire with no corrosion or insulation delamination. Impedance calculated at this instance served as the baseline reference for the successive instances. The next two instances model the high corrosion phase of the electrode. No modification in insulation was included in these two instances. The final four instances model the simultaneous corrosion and insulation delamination phase. A rate of 15 μm per instance was assumed for modeling the high corrosion rate, while a rate of 0.5 μm per instance was assumed for the low corrosion rate. This ratio of 15:0.5 was chosen in accordance with the experimentally measured tungsten corrosion rate ratio of 10,000–20,000:300–700 in the presence and absence of hydrogen peroxide. A constant rate of 2 μm per instance was assumed for the increase in insulation gap width and length.

In Case 2, the insulation crack was modeled as a slit with a width that is proportional to the arc angle and a varying crack length (Figure 8B). Similar to Case 1, the impedance for Case 2 was calculated at seven different instances for the insulation-cracked case. As before, the first instance was a pristine wire serving as the baseline reference for the successive instances, and the next two instances model the high corrosion phase of the electrode, and the final four instances model the simultaneous corrosion and cracking in insulation phase. The same rate of 15 μm per instance was assumed for modeling the high corrosion rate, while a rate of 0.5 μm per instance was assumed for low corrosion rate. A constant rate of 2.5 μm per instance was assumed for the increase in insulation crack length and the width of the crack was set by fixing the arc angle at 15°, 30°, 60°, and 90° for each instance.

**Figure 9:**
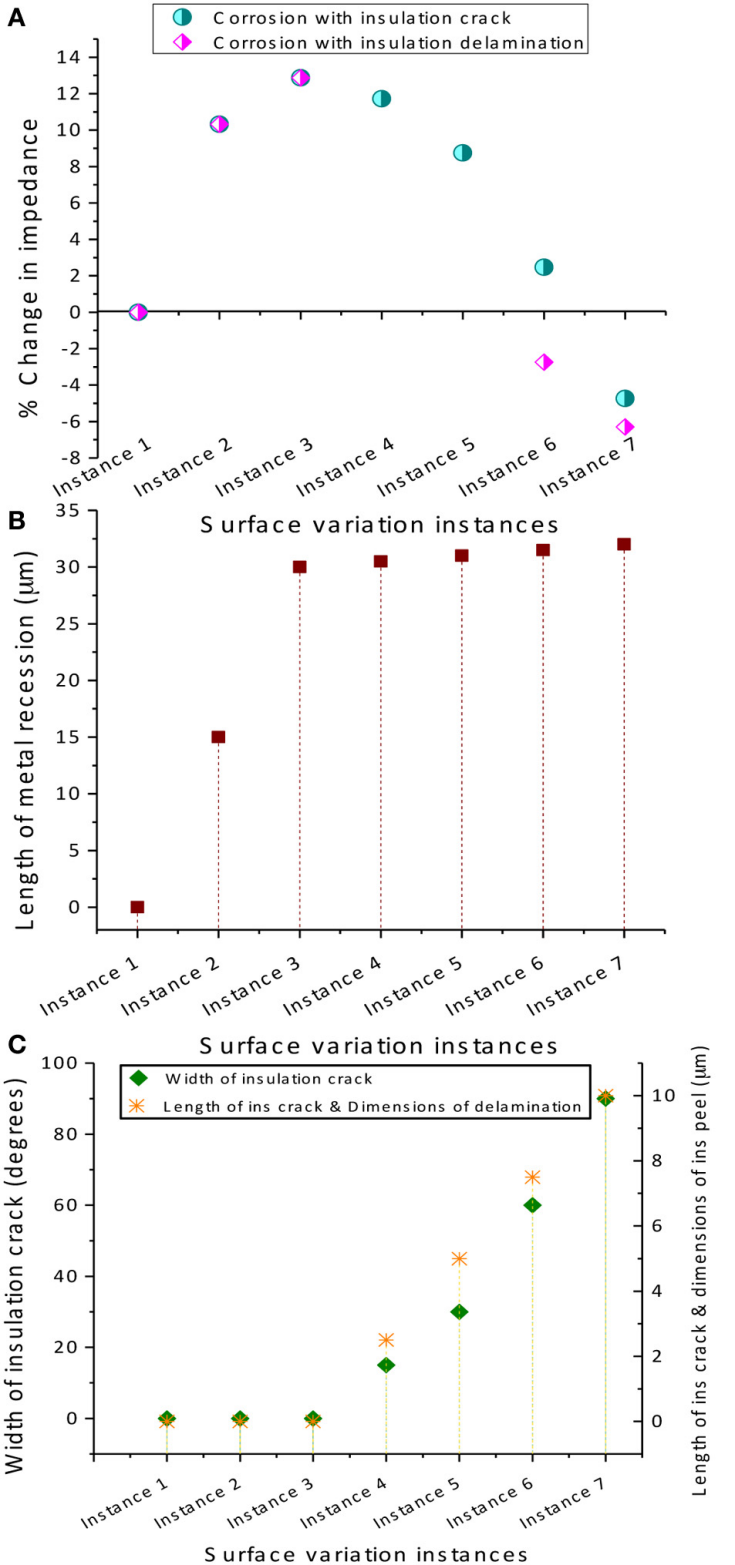
**COMSOL® 3D simulated results. (A)** Percentage change in electrode impedance for corrosion and (Case 1) insulation delamination and (Case 2) insulation cracking at different instances, **(B)** variation in the metal recession length at different instances to model corrosion, and **(C)** variation of insulation crack width and length and delamination dimensions at different instances. Note that a converged solution could not be obtained for instances 4 and 5 for the insulation delamination case since the FEA solver could not generate a mesh for very small variations in geometry.

The COMSOL® simulated percentage changes in resistance for corrosion and (Case 1) insulation delamination and (Case 2) insulation cracking at different instances are given in Figure 9. Also the dimensions of the metal recession modeling corrosion and insulation delamination and cracks are also shown. It can be observed in Figure 9 that the percent change in the electrode resistance increases steadily during the initial high corrosion-rate phase and decreases during the low corrosion and insulation damage phase, modeled either by insulation delamination or by insulation cracking, giving rise to a bell shape or Gaussian profile for both cases. The increase of resistance as a result of the tungsten recessing into its insulation is an interesting finding. However, it can be observed that the increase in resistance is only up to 14% and the decrease in resistance is up to −7% which is nearly one order of magnitude less than the observed *in-vivo* impedance changes.

## Discussion

### Abiotic vs. biotic contributions to impedance magnitude at 1 kHz

#### Abiotic and biotic factors

Before estimating values for elements in the time-varying equivalent circuits, it is important to identify which factor, biotic or abiotic, will most affect the individual circuit elements. The biotic changes at the electrode/tissue interface are encapsulation by extracellular matrix proteins and glial cells brought on by the foreign-body immune response. The abiotic factors that lead to electrode surface morphology are corrosion and insulation deterioration.

The surface area of the recording electrode is constantly changing due to corrosion and insulation delamination. Hence, it is possible that the circuit elements that comprise the neural electrode equivalent circuit model will change over time due to the changing surface area. As seen from the COMSOL® results, the corrosion and subsequent recession of tungsten will cause an increase in the electrolyte resistance, *R*_*e*_, while insulation delamination and cracking will decrease the resistance. Similarly, the Randles circuit parameters, charge transfer resistance (*R*_*ct*_) and CPE coefficient *Q*, are also expected to vary with changes in the surface area. *R*_*ct*_ is inversely proportional to the surface area while *Q* (similar to a capacitance) is directly proportional to area.

The key remaining question is whether the tissue encapsulation circuit will be affected, i.e., will abiotic changes affect biotic changes of the neural electrode? For a first order approximation, one would expect that the encapsulation components *R*_cell_ and *C*_cell_ will be unaffected by changes in surface area since they represent an independent cellular layer. However, it is possible that dynamic changes in abiotic factors such as the surface roughness and area compound the biotic changes due to re-engaging the immune response as fresh electrode surfaces are newly exposed over time. The biotic factors, encapsulation by extracellular matrix proteins and glial cells, will obviously control the circuit elements *R*_*en*_, *C*_cell_, and *R*_cell_. However, *R*_*en*_ will be coupled with abiotic changes since it is also dependent on the surface morphology of the electrode. Moreover, the time-varying void left by the corroding tungsten and/or delaminating/cracking insulation might encourage ingrowth of extracellular matrix proteins and result in a larger encapsulation resistance than would be present for an ideal, non-corroded recording surface. Analysis of the regression suggests a multitude of factors contributing to the magnitude of the impedance change at 1 kHz for the array under study.

#### Analysis of regression

Using the results from the regression and the COMSOL® analysis, we can estimate the circuit elements in the equivalent circuits used to represent the electrode/tissue interface over the chronic implant time for wire 12. According to the COMSOL® results, *R*_*e*_ will initially increase a maximum of 14% from its day one value and decrease to only 7% below this value over time. These changes are small compared to the magnitude of the change of the combined regressed value for series resistance; thus for purposes of the regression analysis, *R*_*e*_ is assumed to be roughly constant and *R*_*en*_ is simply calculated to be the regressed value minus *R*_*e*_ for the Randles, day-one circuit. Nonetheless, the shape of the total impedance change (Figure 4) follows the shape of the electrolyte resistance change (Figure 9).

The real and imaginary parts of the complex impedance contribute to the overall magnitude of the impedance as described in Equation 3

(3) $$Z_{mag}=\sqrt{Z_{r}^{2}+Z_{j}^{2}},$$

where *Z*_*r*_ and *Z*_*j*_ are the real and imaginary parts of the impedance, respectively. Thus it is useful to compare the real and imaginary parts of the different components in the equivalent circuits calculated at 1 kHz. The real and imaginary parts of the parallel combination of *R*_*ct*_ and *Z*_*CPE*_ are termed *Z*_*r*,*j*-interface_ and given in Table 2. The estimated impedance for the parallel combination of *R*_cell_ and *C*_cell_ is termed *Z*_*r*,*j*-cell_ and also given in Table 2 along with estimated values for *R*_*e*_ and *R*_*en*_ from the regression analysis.

**Table 2:** **Real and imaginary parts of impedance of equivalent circuit parameters for wire 12**.

**Impedance (kΩ) at 1 kHz**						
**Day**	***Z*_*r*-interface_**	***Z*_*j*-interface_**	***Z*_*r*-cell_**	***Z*_*j*-cell_**	***R*_*en*_**	***R*_*e*_**
1	3.7	−11.4	0	0	0	21
7	4.2	−9.2	60.0	−24.8	57.4	21
15	3.2	−7.8	22.1	−9.8	32.6	21
31	3.8	−8.3	65.3	−30.1	77	21
60	4.2	−8.6	9.0	−5.8	24.0	21
106	4.4	−8.4	12.2	−3.4	9.0	21
149	5.1	−9.1	16.0	−4.7	11.4	21
205	4.4	−8.7	9.0	−4.7	12.7	21

**Figure 10:**
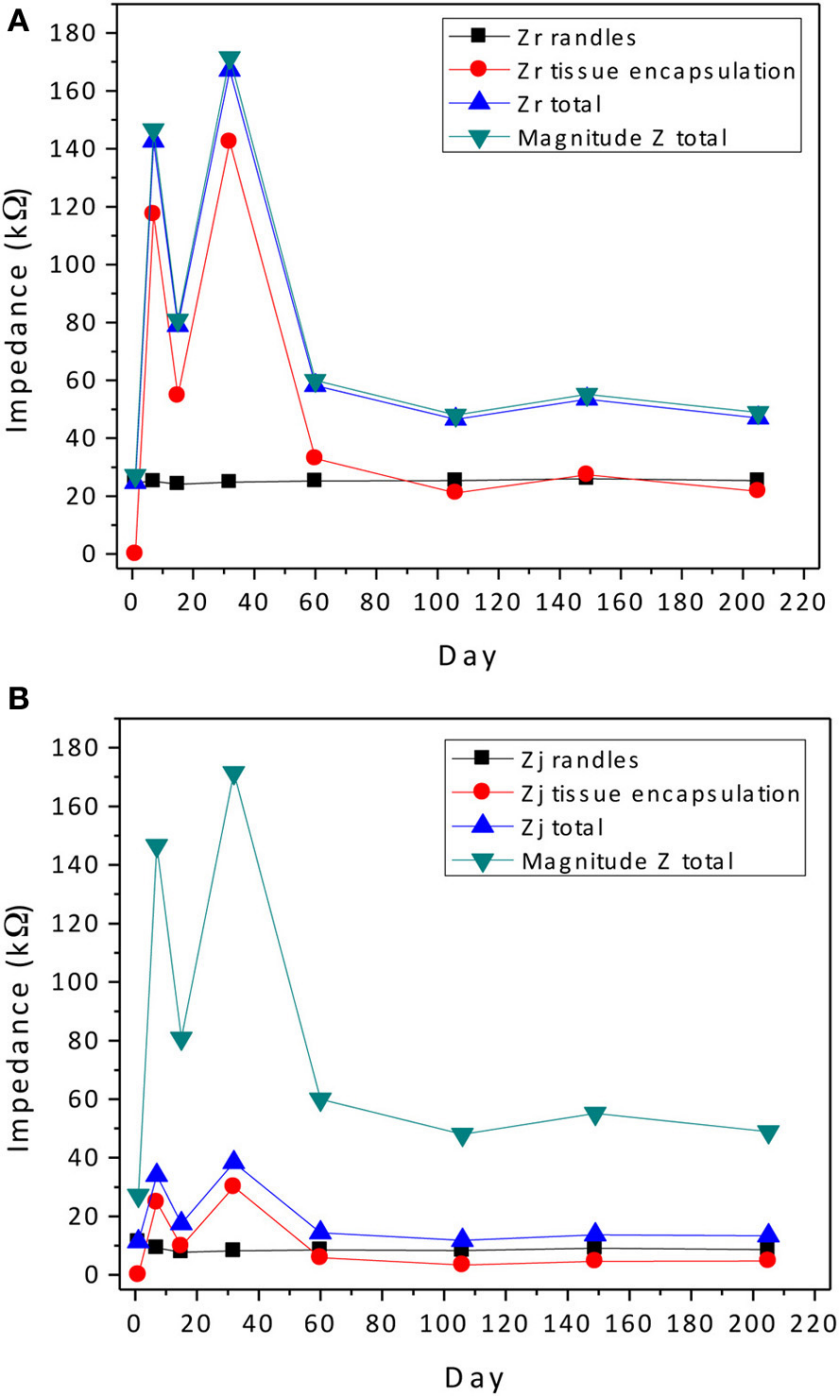
**Contribution of the abiotic and biotic components to the real (A) and imaginary (B) parts of the complex impedance compared to the magnitude of the total impedance**. All impedance values were calculated at 1 kHz.

The contribution of the impedance at 1 kHz for components in the Randles circuit is *Z*_*r*-interface_ + *R*_*e*_ and *Z*_*j*-interface_ for the real and imaginary parts, respectively. The tissue encapsulation contribution is *Z*_*r*-cell_ + *R*_*en*_ and *Z*_*j*-cell_ for the real and imaginary parts, respectively. Since the real and imaginary parts have a non-linear relationship with the impedance magnitude, both are plotted separately and compared against the magnitude of the impedance in Figure 10. Here, key observations can be made. The real part of the impedance is much larger than the imaginary part at 1 kHz. Thus, the magnitude of the complex impedance is dominated by trends in the real part of the impedance as seen in Figure 10A.

There is no *a priori* assumed model for the time dependence of the tissue encapsulation process while the COMSOL® simulation of abiotic changes due to corrosion and insulation delamination/cracking indicate a Gaussian temporal dependence. There are several possible scenarios. Though regression analysis was performed for only one electrode out of the array, because of the similarity of the trends in the complex impedance spectra for the remaining electrodes, the impedance changes are expected to be similar. We surmise that the rise in impedance magnitude, which resulted in an average increase of 100% is dominated by biotic tissue encapsulation factors; however, the coupling or acceleration of the biotic factors by the prolonged foreign body response due to corrosion is unknown. The subsequent fall in the impedance magnitude could be due to both biotic and abiotic factors. Since the tissue encapsulation layer is known to swell in the beginning and then decrease to a more compact layer in the chronic stage, a rise and fall in the impedance magnitude is possible. However, it should not decrease to a value that is lower than the initial value. In at least half of the electrodes in the array under study, the impedance magnitude decreased to a value that was 25–50% lower than the initial value. Therefore, abiotic effects that increased the surface area and perhaps greatly reduced the encapsulation *R*_*en*_ and electrolyte resistance *R*_*e*_ may be significant.

### Limitations to the model

The major source of error in our analysis is the assumption of the equivalent circuit for the *in vivo* electrode/tissue interface. Similar circuit elements were used previously to model the tissue encapsulation (Johnson et al., 2005; Otto et al., 2006; Williams et al., 2007; Lempka et al., 2011). The Randles circuit may not be a good model for an electrode undergoing corrosion where the microstructure contains numerous pores and other surface variations. Incorporation of porous electrode equivalent circuit models could provide a better fit to the experimental data. However, the dominance of the impedance due to tissue encapsulation at 1 kHz makes our estimation appropriate. The uncertainty in the regression was not calculated since a Monte Carlo approach would have been needed to calculate the error in the regressed parameters. Finally, additional impedance data points at higher frequencies would improve the accuracy of the regression analysis. The maximum measurement frequency of 2 kHz was limited by the impedance tester used.

## Conclusions

We examined the complex impedance trends of a 16 channel tungsten microwire array used in a chronic *in vivo* neural recording study. This array had good functional performance throughout the implant duration and its impedance magnitude at 1 kHz showed temporal variability in the first few weeks that on average was Gaussian in shape. Time varying equivalent circuits that model the varying surface morphology and tissue response were shown to exhibit different structure in the complex impedance. To better understand the origin of the temporal change in the real part of the complex impedance, the abiotic electrolyte resistance was modeled in COMSOL® using a time varying geometry that simulated changes in the recession depth of the electrode surface due to corrosion and insulation changes due to delamination and cracking. A similar Gaussian shape in the electrolyte resistance was observed due to the competing effects of recessed electrode surface and increased exposure of the electrode shank area. The latter abiotic mechanism of insulation delamination can result in a lower impedance than the original reference impedance while the biotic mechanisms of tissue encapsulation cannot. Conversely, tissue encapsulation could be responsible for the larger magnitude increase in the electrode impedance initially. It can be concluded that both biotic and abiotic factors play key roles in the observed electrode impedance change in chronically implanted recording electrodes.

To generalize the results of this study, a Gaussian temporal variation of the impedance magnitude at 1 kHz with a rise and fall that changes to upward of 500% represents the effect of the biotic inflammatory response at the electrode/tissue interface and abiotic corrosion and insulation deterioration at the electrode surface that does not necessarily hinder functional performance during the implant duration of 9 months. These results suggest that an electrode array whose impedance magnitude at 1 kHz has very dissimilar temporal variations than the electrodes in this study may indicate that an abnormal abiotic malfunction such as a mechanical failure in the array or severe insulation delamination may have occurred.

### Conflict of interest statement

The authors declare that the research was conducted in the absence of any commercial or financial relationships that could be construed as a potential conflict of interest.
